# Comparison of Postoperative Pain Following One-Visit and Two-Visit Vital Pulpectomy in Primary Teeth: A Single-Blind Randomized Clinical Trial 

**DOI:** 10.22037/iej.v13i1.18205

**Published:** 2018

**Authors:** Elham Farokh-Gisour, Masoud Parirokh, Marjan Kheirmand Parizi, Nouzar Nakhaee, Masoumeh Aminizadeh

**Affiliations:** a *Department of Pediatric Dentistry, Dental School, Kerman University of Medical Sciences, Kerman University of Medical Sciences, Kerman, Iran; *; b *Oral and Dental Disease Research Center, Dental School, Kerman University of Medical Sciences, Kerman, Iran; *; c *Student Research Committee, Kerman University of Medical Sciences, Kerman, Iran; *; d * Neuroscience Research Center, Kerman University of Medical Sciences, Kerman, Iran; *; e * Dental Research Center, Department of Pediatric Dentistry, Zahedan University of Medical Sciences, Zahedan, Iran*

**Keywords:** Children, One-Visit, Pain, Postoperative, Primary Teeth, Pulpectomy, Two-Visit

## Abstract

**Introduction:**

The aim of this study was to compare post-operative pain following one-visit pulpectomy and placing stainless steel crown (SSC), with two-visit treatment (performing pulpectomy at the first visit followed by placing SSC at the next visit one week later) in vital pulp of primary molars with carious involvement.

**Methods and Materials:**

In this randomized clinical trial, 100 children aged 6-12 years with a carious primary molar tooth in need of pulpectomy were randomly divided into two groups of 50 each. In one-visit group, pulpectomy and placement of SSC were carried out at the same appointment. In two-visit group, pulpectomy of root canals was carried out at the first visit and placement of SSC was performed at the second visit one week after the first appointment. Post-operative pain was recorded using visual analogue scale (VAS) during one week after each treatment visit.

**Results:**

No significant difference was found in the mean age and gender distribution between the two groups (*P*˃0.05 for both comparisons). Findings revealed that in the two-visit (pulpectomy) group during first three days and 4-7 days after the first treatment appointment, pain felt by the children was significantly lower than that felt by the one-visit group at the same time period (*P*˂0.0001 for both comparisons). Moreover, children in two-visit (pulpectomy) group consumed significantly lower amount of analgesics than those in the one-visit group (*P*<0.0001).

**Conclusion:**

No significant difference was found between pain felt by children during the first three days following one-visit pulpectomy and placement of SSC at the same appointment. Therefore, one-visit treatment of vital primary tooth is recommended.

## Introduction

Pain is an unpleasant multidimensional experience which comprises strong sensory and cognitive components [1]. The prevalence of postoperatice pain in children has reported to 65.6% in dental procedures [2]. Pain can also limit daily life activities, so it can be regarded as an important public health issue [3]. Taking control of pain is of high importance in the field of pediatrics dentistry because the concept of dental care is stabilized in people’s mind during childhood [4]. It is also possible that, if children suffer from pain during the restorative procedures or when they undergo dental surgery, their future may be damaged as dental patients [5]. 

Fear of pain following dental treatment can cause parents to prevent their children from visiting dentists for dental care [6]. Children often lack the needed cognitive and linguistic skills to talk about their physical discomfort and pain intensity [7]. Pain is a subjective phenomenon and varies from one person to another [7, 8]. As a result of less verbal communication and differences in the level of maturity and understanding of questions, it is more difficult to evaluate and measure the level of pain in children compared to adults [8]. Therefore, the gold standard for pain assessment can be expressed on the basis of individual reports [7-9].

The main purpose of pulp treatment in primary teeth is to maintain and preserve them as the important parts of the dental arch to provide the ability to chew and speak, to maintain required space for the permanent teeth to replace the primary teeth and to effectively prevent damages caused by tooth decay [10]. To achieve these important goals, dentists usually use vital pulpotomy technique. Nevertheless, in some patients with irreversible pulpitis, or with necrotic radicular canals, pulpotomy technique was not successful and therefore another technique known as partial or complete pulpectomy treatment is indicated [10]. In fact, this technique is a radical treatment for the prevention of loss of the primary teeth which results in preservation of the dental arch length, creating adequate space for the eruption of permanent teeth and preventing pressure on the premolars and molars [11]. Moreover, the pulpectomy technique is an optimal and effective way to preserve primary molars [12, 13]. In full pulpectomy, the inflamed or necrotic pulp tissue is removed and then the evacuated area will be filled with absorbent paste [11]. However, the experience gained from using this technique shows that this method enjoys a lot of therapeutic success [14, 15]. Another limitation of this method is that full pulpectomy surgery causes considerable pain and this leads to a significant decrease in patient satisfaction. On the contrary, whether the number of visits required using this technique affects the results or not, has not yet been evaluated and the questions remain unanswered [16]. Therefore, the aim of this clinical trial study was to compare the level of post-treatment pain between one-visit and two-visit pulpectomy treatment.

## Materials and Methods

This randomized clinical trial was approved by the Ethics Committee of Kerman University of Medical Sciences and was recorded at Iranian Registry of Clinical Trials (IRCT) center (IRCT2016122231473N1). Moreover, given α=5% and power of 80%, each group required a sample size of 50 patients.

A total 0f 100 participants met the qualifications required to enter this prospective, randomized and single-blind study. All the participating patients received treatment at the postgraduate clinic of the Endodontic Department of Kerman Dental School, Iran (from August 2016 to February 2017). To randomize the patients, each patient was assigned a number. The numbers in each group were written on paper, and each one was kept in a separate sealed opaque envelope. Each patient was required to select one of the envelopes and was assigned to one of the groups according to the number. It should be noted that after the nature of the procedure and the possible risks and benefits was explained, written informed consent was obtained from all participants. To assess pain levels, a visual analogue pain scale (VAS) was utilized. Furthermore, the other form was applied to record analgesic consumption after they felt pain. The VAS was explained to the parents and children, instructing them how to use it. They were asked to fill the VAS before local anesthesia was administered to rate their preoperative pain. 

The following inclusion and exclusion criteria were used. Inclusion criteria were as follows: 1- Vital primary molar teeth with active bleeding at the time of access cavity preparation. 2- The teeth should not have any sign of clinical and radiographic evidence indicating pulp necrosis. 3- Root resorption should be less than two-third of the root. 4- The restorable tooth should be curable. 5- The children should be cooperative. 6- There must be at least one molar with irreversible pulpitis signs such as sensitivity to coldness or heat. 7- The patients should require pulpectomy treatment. On the other hand, exclusion criteria were: 1- Suffering from a systematic disease. 2- There must be a follicular or dentigerous cyst beneath the primary molar teeth. 3- There must be at least one-third of pathological decay in the root caused by fistulous sinus track. 4- There must be more than two-thirds decay of the root length. 5- Lack of tooth isolation. 6- Advanced internal resorption. 7- Periapical radiolucency covering permanent tooth bud. 8- Inability to complete questionnaire by the child and parents within one week after the treatment. 9- Existence of cases like internal resorption, widening of the PDL, radiolucency, degeneration and calcification before beginning the study [16].

Patients were randomly divided into two one-visit (a combination of pulpectomy and placing stainless steel crown (SSC) in one-visit) and two-visit treatment (performing pulpectomy at the first visit followed by placing SSC at the next visit one week later) groups. One-visit treatment was performed as follows: Teeth were anesthetized using 2% lidocaine 1:80000 (Daroupakhsh Co, Tehran, Iran) followed by isolation under rubber dam. Tooth caries were removed using round fine finishing bur (Tyzkavan Co, Tehran, Iran).

**Figure 1:**
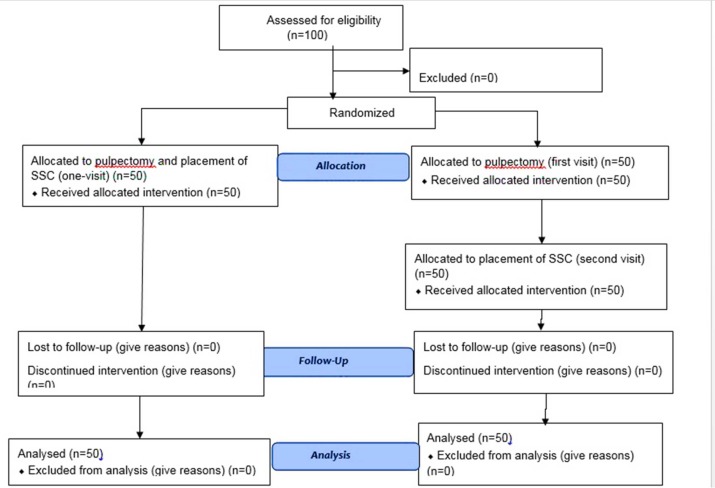
Diagram of patient flow chart

After removing the pulp tissue, if the bleeding did not stop, then under certain circumstances for pulpectomy, barbed broach was utilized to remove pulp tissue from root canals. A H-file (Maillefer, Ballaigues, Switzerland) was used to remove the remaining pulp tissue. After removing the pulp from the root, the canals were irrigated with 2% chlorhexidine using syringes. This was an important step in decreasing microbial contamination. Then canals were dried using paper cone. When the bleeding was stopped and the canals got dried, the root canal walls were covered using paper cones dipped with a mixture of diluted zinc oxide-eugenol (ZEO) pulp. Then a thick mixture of medical paste in the form of small granules was compressed into the canals. Root canal pluggers were used to press the materials in the root canals. Radiography was used to assess the internal structure of the obturation. Eventually, the tooth was restored using SSC. Pain and analgesic consumption questionnaire was completed by the children and their parents within a week after the treatment. 

Two-visit treatment was conducted as follows: in the first visit, pulpectomy treatment was performed entirely by specialized assistant and the questionnaire was completed by the children and their parents within a week after the treatment. In the second visit, the tooth was restored using a SSC by a pediatric dentist (Blinded) and when the treatment was completed; the children and their parents recompleted the questionnaire within a week. So, the process of pulpectomy treatment was carried out entirely by a specialized pediatric assistant and SSC was bonded to the tooth by the plan executor (pediatrician). In order to control the pain at home, the parents were instructed to administer 325 mg of acetaminophen (Daroupakhsh Co, Tehran, Iran) to their children every six hours and record the numbers of pills taken by the children in the questionnaire. 

**Figure 2:**
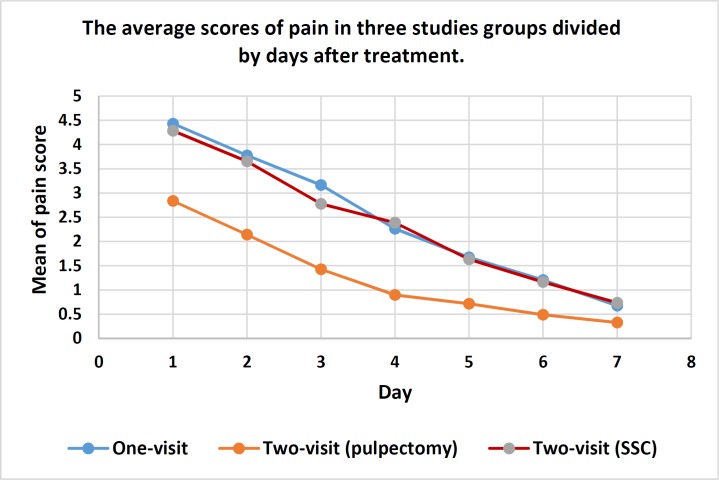
The average scores of pain in one-visit, two-visit (pulpectomy) and two-visit (SSC) groups divided by days after treatment

Participants were given two forms to complete. The first one was VAS form to record the severity of pain from 0 to 9 during a week after the first treatment appointment, and the other form was used to record analgesic consumption after they felt pain [17-19]. VAS questionnaire is one of the most reliable self-report measuring tools used for children to express their pain. It has been widely employed in scientific studies related to pain and its validity and reliability have been confirmed repeatedly.

***Statistical analysis:***

Continuous variables were displayed as mean ± standard error of mean (SEM), and qualitative variables as numbers (percentage). Chi-square test was used to compare the qualitative variables. Independent Sample t-test was used to compare the mean of pain between groups. The *P*<0.05 were considered statistically significant. Statistical analysis was performed using SPSS version 21 for windows (SPSS Inc., Chicago, IL).

## Results

Having met the qualifications for participating in the study, none of the selected one-hundred patients was excluded from the research, thereby continuing the course of steady as demonstrated in Figure 1.

Table 1 contains demographic characteristics of participants. Girls (*n*=26) and boys (*n*=24) accounted for 52% and 45%, respectively, of the 50 participants in the one-visit group. In the two-visit group, girls (*n*=24) and boys (*n*=26) constitute 45% and 52%, respectively, of the all participants. No significant difference was found in the mean age and gender distribution between the two groups.

Table 2 illustrates the comparison of the pain mean scores as well as analgesic consumption between one-visit and two-visit (pulpectomy) on the one hand, and one-visit and two-visit (SSC) groups, on the other. Findings of the study revealed that in the two-visit (pulpectomy) group during the first three days after the first appointment, pain felt by the patients was significantly lower than that felt by the one-visit group at the same time period and the rest of the study period (i.e. 4-7 days) (*P*<0.0001 for both comparisons). However, no significant pain difference felt by children was observed between the two-visit (SSC) and one-visit groups at both the first three days after the first appointment and the rest of the study period (*P*=0.396 and *P*=0.846, respectively). Children in two-visit (pulpectomy) group consumed significantly lower amount of analgesics than those in the one-visit group (*P*<0.0001). However, no significant difference was found in the mean number of analgesics between two-visit (SSC) and one-visit groups (*P*=0.172).

Linearity graph of pain score divided by days after treatment within each three groups is shown in Figure 2. Participants in each group reported a regular decrease regarding the average pain during the seven days after treatment.

There was no significant difference regarding the extended to which the one-visit group (*n*=42, 84%) and two-visit group (*n*=44, 88%) require analgesics (*P*˃0.05). However, children in the two-visit (pulpectomy) group (28 (56%)) had significantly lower need to analgesics need than those in the one-visit and two-visit (SSC) groups (*P*˂0.05 for both comparison).

## Discussion

Based on the present findings, pulpectomy of primary teeth showed significantly lower pain compared to placement of SSC (*P*<0.05). Nowadays, studies related to pain are of particular importance in the field of healthcare around the globe. Despite the great progress that has been made in dentistry, post-treatment pain in tooth root is still an important complication after the treatment. Preventing or minimizing post-treatment pain is an important issue which has been investigated in different studies [20]. It has been approved in dentistry that using analgesics before administering treatment will significantly decrease post-operative pain [21-23]. In comparison to the children, a lot of studies have been carried out among the adults which are related to the benefits of analgesics taken before and after dental treatment; but this topic has not been studied among children and there is also very little research in this area [24]. Therefore, the aim of this clinical trial was to compare the level of pain following two-visit and one-visit pulpectomy of vital primary teeth among 100 children aged 6 to 12 visiting the Department of Pediatrics, Dentistry School of Kerman University of Medical Sciences.

Prior to administering analgesics before and after the operation, dentists should know which method causes less pain in children. Dental pain and analgesics consumption is much more common after dental treatments like tooth crowning, root canal treatment and extraction. The edge of the crown can put a lot of pressure on the gum and cause gingivitis and therefore results in severe pain. It is also possible that plunger cusps exert pressure on the teeth and cause pain; which is due to the difficulty to fully adjust the occlusion after tooth crowning. Nevertheless, there is a strong relationship between the type of treatment and tooth pain after medical treatments, it is still possible that some younger children may feel the pain but fail to detect the exact location of the pain [25].

In their study, Ashkenazi *et al.* showed generally that 38% of the children participating in the study suffer from post-treatment pain [25]. Fifty-six percent of the 39 children participating in the one-visit treatment (pulpotomy with crowning) suffer from post-treatment pain. Moreover, eight children also received pulpotomy treatment without their teeth being crowned and 62% of them complained from post-pulpotomy pain within the first 24 h after receiving the treatment; 42% of them had received analgesics and did not show any significant difference in comparison to one-visit treatment. The results of their study contradicted the average pain in the children participating in two-visit treatment (the first 3 days after treatment) compare to children participating in one-visit treatment (the first 3 days after treatment). On the other hand, in tooth crowning without pulpotomy, despite taking analgesics (63%), 66% of the participants reported post-tooth crowning pain which had significant differences compared to one-visit treatment. Nevertheless, the average pain in the SSC group of present study (the first three days after treatment) did not show any significant difference compare to one-visit treatment (the first three days after treatment). There are some differences between the present study and previous studies that are related to this topic. First, the study design was a single-blind randomized clinical trial but previous studies were cross-sectional, retrospective and the samples were not randomized [20]. Second, in previous studies, the researchers conducted a telephoning survey and ask the parents to complete the questionnaire and grade the pain (24 hours after the treatment)[25]; however, in the current study, the children graded their pain in the presence of their parents. Third, the sample sizes of the previous studies were smaller than the present study [23, 26]. 

**Table 1:** Demographic characteristics of participants

		**One-visit (n=50)**	**Two-visit (n=50)**	***P*** **-value**
**Sex**	Male (%)	24 (48)	26 (52)	0.73
	Female (%)	26 (52)	24 (48)	
**Age (year)**		8.18 ± 1.18	8.26 ± 1.12	0.68

**Table 2:** Comparison of pain mean scores and analgesics consumption among groups

	**One-visit (pulpectomy+SSC)**	**Two-visit (pulpectomy)**	***P*** **-value**	**One-visit (pulpectomy+SSC)**	**Two-visit (SSC)**	***P-*** **value**
**Pain (1-3 day)**	3.77±0.17	2.14±0.15	<0.0001*	3.77±0.17	3.55±0.18	0.396
**Pain (4-7 day)**	1.43±0.14	0.6±0.09	<0.0001*	1.43±0.14	1.47±0.15	0.846
**Analgesics consumption **	2.02±0.21	0.94±0.14	<0.0001*	2.02±0.21	-	0.172

Mustafa *et al.* [27] revealed that there is a relationship between the types of treatment and post-operation pain in children. Twenty-five children participated in their study and underwent pulpectomy treatment. Their results indicated that the average pain is higher within 6 hour after treatment compare to the first two hours. However, in the present study, we observed that there was a decrease in pain after both one-visit and two-visit pulpectomy treatments; meanwhile the average post-treatment pain in two-visit treatment was significantly lower than post-treatment pain in one-visit treatment (the first 3 days after treatment).

In their study, they concluded that 6 hours after tooth crowning, a decrease in pain among all 10 children participating in the study could be observed [25]. However, in the present study, after bonding SSC to the tooth, there was no significant difference in the average pain compared to one-visit pulpectomy treatment.

Using different methods of statistical analysis could be the reason of non-identical results. Different times were evaluated in Mustafa *et al.* [27] study, however, in the present study, the average pain was statistically analyzed during the first three days after the intervention. The average pain was statistically reanalyzed four days later to draw a more realistic picture of the pain and to calculate the required dose of analgesics after intervention.

The availability of evidence about the state of the tooth before endodontic treatment as well as other local physical factors that can affect the postoperative endodontic pain can help in interpreting the post-treatment pain. In this regard, de Andrade *et al.* showed that spontaneous pain and insensitivity to the pain before the operation has a significant effect on creating pain after the operation [26]. Also in another study, researchers revealed that children who had pre-operative pain or dental abscess, after treatment complained of more pain than children who had no preoperative pain [25].

## Conclusion

Taken together, pain felt by the children in two-visit (pulpectomy) children was significantly lower than one-visit and two-visit (SSC) groups during first three days after the first appointment. However, pain felt by children during the first three days following one-visit pulpectomy and placement of SSC at the same appointment was almost the same. Therefore, one-visit treatment of vital primary tooth is recommended. Due to the complex etiology of pain and limited experience of aggressive treatment among children in this age range, predicting and healing the pediatric pain during the process of treatment and also after dental treatments, should be an integral part of child care.
